# Rhabdomyolysis: a narrative review

**DOI:** 10.1055/s-0046-1824437

**Published:** 2026-06-23

**Authors:** Celia Harumi Tengan, Acary Souza Bulle Oliveira, Alzira Alves de Siqueira Carvalho, Cezar Daniel Snak de Souza, Claudia Ferreira da Rosa Sobreira, Edmar Zanoteli, Helga Cristina Almeida da Silva

**Affiliations:** 1 Universidade Federal de São Paulo, Escola Paulista de Medicina, Departamento de Neurologia, São Paulo SP, Brazil.; 2 Centro Universitário Faculdade de Medicina do ABC (FMABC), Faculdade de Medicina, Departamento de Neurologia, São Paulo SP, Brazil.; 3 Universidade Federal de São Paulo, Escola Paulista de Medicina, Departamento de Anestesiologia, São Paulo SP, Brazil.; 4 Universidade de São Paulo, Faculdade de Medicina de Ribeirão Preto, Departamento de Neurociências e Ciências do Comportamento, Ribeirão Preto SP, Brazil.; 5 Universidade de São Paulo, Faculdade de Medicina, Departamento de Neurologia, São Paulo SP, Brazil.

**Keywords:** Glycogen Storage Disease, Malignant Hyperthermia, Mitochondrial Myopathies, Muscular Dystrophies, Myositis, Myotonic Disorders, Rhabdomyolysis

## Abstract

Rhabdomyolysis is the acute necrosis of striated skeletal muscle, with release of its constituents into the extracellular space and circulation. Acute muscle pain (myalgia), weakness, and edema are associated with serum levels of the muscle enzyme creatine kinase (CK) above 1,000 IU/L or 5 times the upper limit of the normal value. This narrative review provides an overview of the clinical and laboratory findings, etiology, treatment, and prevention of rhabdomyolysis in humans. Additionally, we highlighted the main syndromes and muscular disorders associated with rhabdomyolysis: 1. Clinical syndromes: neuroleptic malignant syndrome, serotoninergic syndrome, sympathomimetic syndrome, malignant hyperthermia, and anesthesia-induced rhabdomyolysis; and 2. Myopathies: toxic myopathies, idiopathic inflammatory myopathies, muscular dystrophies, ion channel diseases, glycogen storage diseases, fatty acid beta oxidation defects, and mitochondrial myopathies.

## INTRODUCTION

Rhabdomyolysis is the acute necrosis of striated skeletal muscle, with release of its constituents into the extracellular space and circulation. Acute muscle pain (myalgia), weakness, and edema are associated with serum levels of the muscle enzyme creatine kinase (CK) above 1,000 IU/L or 5 times the upper limit of the normal value. 1 Dark urine (myoglobinuria) and acute kidney injury (AKI) are hallmarks of severity. Exclusion criteria for rhabdomyolysis include elevated CK due to heart disease (e.g., myocardial infarction), brain disease (e.g., stroke), or chronic muscular disorders (e.g., Duchenne muscular dystrophy).

In Brazil, the incidence of rhabdomyolysis in a tertiary hospital was 3.8% of all inpatients over 18 months, with a mortality rate of 40%. 2 In the United States, 26,000 cases of rhabdomyolysis are reported annually, with an incidence of 1:1,500 pediatric consultations and a recurrence rate of 5 to 11%. 3

Rhabdomyolysis is underdiagnosed. As it is of interest to different specialties, their integration is fundamental to promoting early diagnosis, targeted investigation, and the prevention of complications. This narrative review presents the opinion of Brazilian experts (six neurologists/one anesthesiologist) who reviewed the relevant literature of their specific section to provide an overview of rhabdomyolysis. Each section highlights the essential material for a broad view, with basic introductory information to attend to the generalist, as well as useful details for specialists from areas such as neurology, anesthesiology and intensive therapy. For some sections, linked to situations commonly associated with rhabdomyolysis, reviews covered most of these objectives. In situations rarely associated with rhabdomyolysis, case reports/series were employed.

### CLINICAL AND LABORATORY PRESENTATION

Rhabdomyolysis severity is variable, ranging from fatal outcomes to around 50% of asymptomatic recognized cases. 4 However, this diagnosis may go unnoticed, especially in unconscious patients or during the postoperative period. Sometimes, rhabdomyolysis is diagnosed only after the onset of AKI. It is crucial to search for signs of weakness/swelling in muscle groups, dark urine (pigmenturia), and muscle enzymes abnormalities. Less than 10% of the patients present the classic triad of pain, weakness, and dark urine. 5

Rhabdomyolysis is associated with elevated serum levels of muscle components, including myoglobin, creatine kinase (CK), lactate dehydrogenase, transaminases, aldolase, lactate, electrolytes (potassium, phosphorus), and uric acid. 4 6 Myoglobin, a low molecular weight heme protein, found in cardiac/skeletal muscle, is responsible for transporting/storing oxygen. 3 The serum peak of myoglobin occurs earlier than that of CK, typically rising within 1 to 3 hours, peaking at approximately 12 hours, and normalizing within 24 hours. Creatine kinase, an enzyme that transfers phosphate from phosphocreatine to adenosine diphosphate (ADP), contributing to energy production, increases within 2 to 12 hours, peaks between 24 and 72 hours, and typically normalizes within 5 days. 4 6 Consequently, serial measurement of CK/myoglobin is recommended for monitoring of rhabdomyolysis. Myoglobinuria correlates with urinary/serum levels respectively above 100 mg/dl and 300 ng/ml, 7 with an amber-colored serum and dark urine. Myoglobinuria may be suspected when urine analysis detects hemoglobin in the absence of red blood cells in the urinary sediment. 4 6

Morbidity/mortality associated with rhabdomyolysis are related to the occurrence and intensity of complications, which peak between 72 and 96 hours. 4 The main complications are arrhythmias, AKI, compartment syndrome, and disseminated intravascular coagulation (DIC). Arrhythmias are associated with hyperkalemia and/or cardiac necrosis due to direct injury caused by the triggering agent of rhabdomyolysis. Myoglobin deposition in renal tubules causes acute tubular necrosis and AKI, which occurs in 13 to 50% of cases and can increase mortality from 8 to almost 60% among patients undergoing dialysis. 4 5 This variability reflects patient settings (emergency vs. intensive care), etiology (trauma vs. drug-induced vs. secondary to neuromuscular disease) and severity. The renal insult may be aggravated by acidosis and hypovolemia, resulting from fluid sequestration in swollen muscles. McMahon et al. (2013) developed a matrix to predict the risk of AKI/death after rhabdomyolysis, considering the variables age, sex, initial serum creatinine/calcium/CK/phosphorus/bicarbonate values, as well as excluding myositis/seizures/syncope/exercise, and statins use. In the high-risk group (score >10; range 0–26), 61.2% of patients died or required dialysis 8

Compartment syndrome occurs when edematous necrotic muscle tissue is compressed against the overlying fascia, leading to increased pressure in the muscle compartment, which amplifies the initial necrotic process by damaging vascular bundles (secondary ischemic lesions with muscle infarction) and motor and sensory nerves (neuropathies). Imaging studies (magnetic resonance imaging [MRI] and ultrasound) can show edema in the affected muscle distribution. 4 This syndrome is also known as the ‘five-Ps’ disease due to the presence of pain, pallor, pulselessness, paresthesias, and paralysis. Without timely treatment, amputation of the affected limb may be necessary.

Other complications associated with rhabdomyolysis include hypocalcemia, hepatitis, and DIC. Diagnosing liver inflammation during rhabdomyolysis is a challenge because transaminases are also released by the skeletal muscle, and their use in monitoring liver disease is compromised. In these cases, formulas can be used to estimate the proportion of the increase in transaminases that would be due to rhabdomyolysis. 9 Additionally, liver disease can be suspected if high levels of gamma-glutamyl transferase, bilirubin, or alkaline phosphatase are present, as well as changes in liver imaging tests. Disseminated intravascular coagulation, resulting from pro-coagulant factors in necrotic muscle tissue and unopposed activation of the coagulation cascade, can lead to subsequent generalized hemorrhagic and thrombotic phenomena and is associated with an increased risk of AKI/death, making platelet count and coagulation tests (prothrombin time, partial thromboplastin time, fibrinogen, and D-dimer levels) necessary.

### PATHOGENESIS

The various causes of rhabdomyolysis share a common pathophysiological mechanism that begins with energy reserve depletion (adenosine triphosphate [ATP]) and intracellular calcium accumulation. Impairment of the sodium-potassium pump leads to cellular edema, activation of phospholipases/proteases, cell death, and secondary inflammation. 10 Depletion of energy reserves occurs mostly in hypermetabolic syndromes, hyperthermia, sustained muscle contraction, and metabolic myopathies. Lesions of the cell membrane or defects in intracellular channels increase intracellular calcium. Regardless of the etiology, rhabdomyolysis can be precipitated by febrile illnesses or physical activity, situations in which hyperthermia and the release of pro-inflammatory substances can decompensate preexisting diseases, leading to acute muscle necrosis. 10 For instance, malignant hyperthermia (MH) crises are preceded by strenuous exercise or fever episodes 72 hours before. 11

### ETIOLOGY

**Figure 1:**
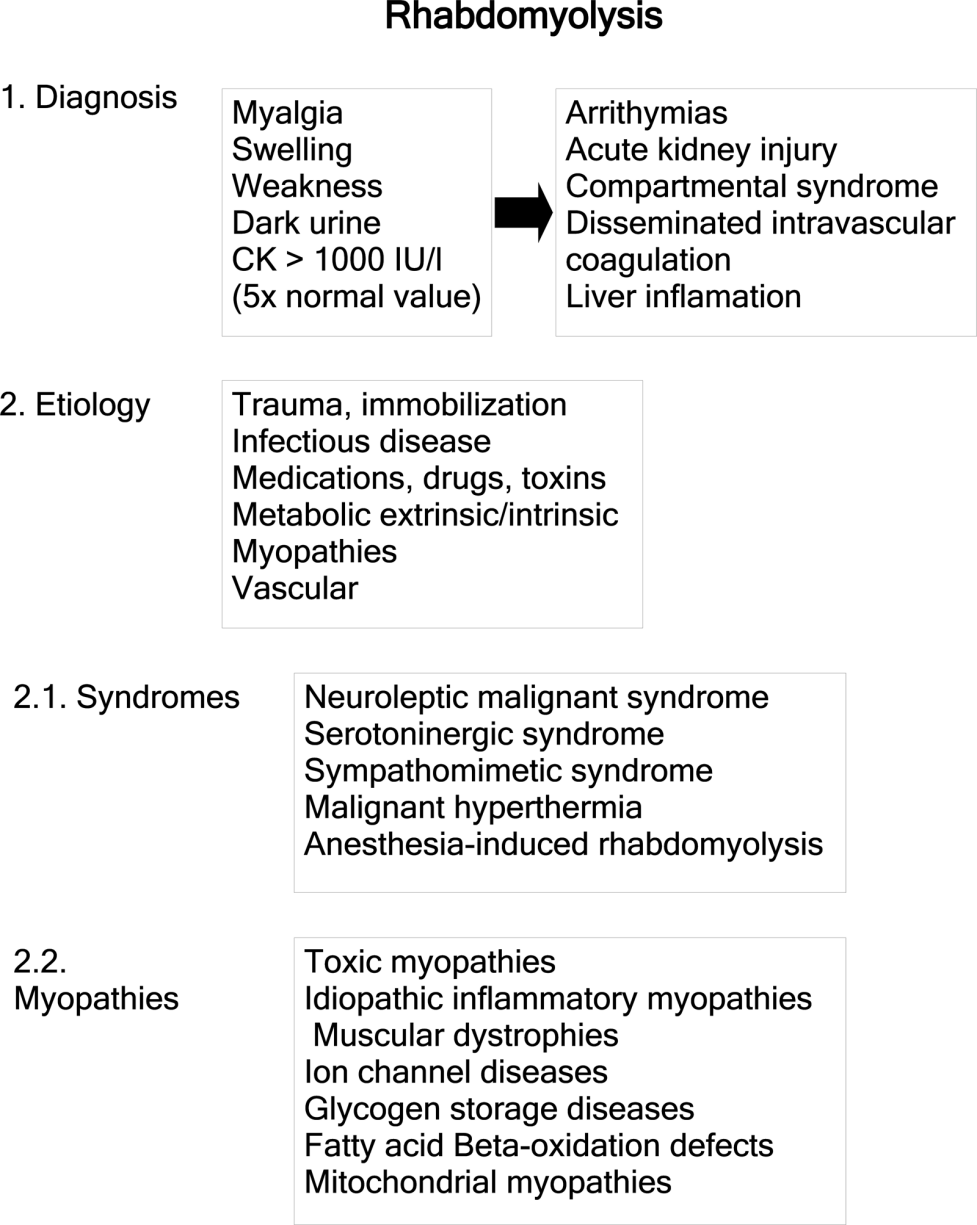
Main points of rhabdomyolysis diagnosis.

The etiology of rhabdomyolysis remains unknown in up to 50% of cases. In the remaining cases, the most common cause is muscle crushing ( Table 1 , Figure 1 ) resulting from trauma and/or prolonged immobilization, particularly in overweight patients and those anesthetized for extended periods of time (e.g., bariatric surgeries or nephrectomies), especially when positioned prone or laterally. 4 5 6 In a previous study, 83.3% of patients who had surgeries lasting more than 7 hours developed rhabdomyolysis. 12

**Table 1:** Rhabdomyolysis etiology

Etiology	Subgroups/mechanism	Examples
Crushing	Trauma	Landslip, car crash
	Immobilization	Head trauma, stroke, seizures, alcohol and/or drug use, surgery
Medications	Hypokalemia	Corticosteroids, diuretics, theophylline, amphotericin B
	Respiratory chain block	Propofol
	HMGCR inhibition	Statins
	Immunosuppressants	Azathioprine, azacitidine
	Neuroleptic malignant syndrome	Neuroleptics: haloperidol, quetiapine, risperidone
	Malignant hyperthermia	Succinylcholine, halogenated agents
	Anesthesia-induced rhabdomyolysis	Succinylcholine
	Serotonin syndrome	Serotonin recaptation inhibitors, ondansetron, opioids, methylene blue
	Others	Barbiturates, benzodiazepines, antihistamines, caffeine
Drugs	Licit drugs	Alcohol
	Illicit drugs	Stimulants (amphetamines, cocaine), opioids (heroin), inhalants (toluene), MDMA
	Poisons	Carbon monoxide, strychnine
Myotoxins	Toxins	Fish, bee/wasp stings, spider/snake venom
	Bacteria	*Staphylococcus* , *Borrelia* , *Legionella* , *Streptococcus* , *Vibrio* , *Salmonella* , *Mycoplasma* , *Neisseria* , *Leptospira* , *Escherichia coli* , *Rickettsia* , *Listeria* , *Clostridium*
	Protozoa	*Trichinella* , *Plasmodium*
	Fungi	*Candida* , *Aspergillus*
	Viruses	Influenza, parainfluenza, adenovirus, human immunodeficiency virus, coxsackie, enteric cytopathic human orphan, cytomegalovirus, herpes, varicella-zoster, Epstein-Barr, chikungunya, coronavirus disease 2019, dengue
Metabolic extrinsic	Exercise/hypermetabolism	Heatstroke (marathon, military service), infectious tetanus, delirium tremens, status epilepticus, dystonia, electrical current injury
	Temperature changes	Hyperthermia, hypothermia
	Endocrinopathies	Diabetes mellitus/insipidus, thyroid/pituitary diseases, hyperaldosteronism, adrenal insufficiency
	Hydro-electrolytic alterations	Acidosis, hypokalemia, hypocalcemia, hypophosphatemia, hypernatremia, hyperosmolarity, water intoxication
Metabolic intrinsic	Metabolic myopathies	Glycogen storage diseases, fatty acid Beta oxidation defects, mitochondrial myopathies
Myopathies	Congenital myopathies	Ryanodinopathies
	Dystrophies	Duchenne and Becker muscular dystrophies
	Inflammatory myopathies	Dermatomyositis, sporadic inclusion body myositis, immune-mediated necrotizing myopathies
Vascular	Arterial/venous obstruction	Tourniquets, extracorporeal circulation, transplants, vascular prosthesis, hemoglobinopathies

Abbreviations: HMGCR, 3-hydroxy-3-methylglutaryl-coenzyme A reductase; MDMA, 3,4-methylenedioxymethamphetamine (commonly known as “ecstasy).

Rhabdomyolysis may be triggered by substances that are myotoxins or induce phospholipases or metabolic disruption, such as medications, drugs, and toxins ( Table 1 ). 5 Medication-induced rhabdomyolysis occurs in 1:100,000 individuals, and is more common in men, those over 65 years of age, and patients taking concomitant medications metabolized by the hepatic cytochrome P450 enzyme system (CYP) or additional medications that impair the CYP (e.g. amiodarone, antiretrovirals, clarithromycin, fibrates, itraconazole, verapamil).

Nearly 200 medications are associated with rhabdomyolysis, 13 through various mechanisms, such as hypokalemia, electron transport chain blockade, and 3-hydroxy-3-methylglutaryl- coenzyme A reductase (HMGCR) inhibition ( Table 1 ). Legal and illegal drugs can cause rhabdomyolysis ( Table 1 ). Additionally, some medications/drugs are involved in clinical syndromes linked to rhabdomyolysis, such as neuroleptic malignant syndrome (NMS), MH, anesthesia-induced rhabdomyolysis (AIR), and serotonin syndrome. Medications/drugs that interact with dopaminergic, serotonergic, and catecholaminergic systems trigger rhabdomyolysis by dysregulating the hypothalamic thermoregulatory center and leading to excessive heat production due to muscle rigidity/contraction.

Toxins associated with rhabdomyolysis include biological substances that cause injury through direct or immune-mediated mechanisms ( Table 1 ), such as fish and seafood contaminated with toxins from algae/cyanobacteria (Haff's disease). 14 Rhabdomyolysis may also be linked to infections/toxins from bacteria, protozoa, fungi, and viruses. 7 15 16 17 18

In metabolic rhabdomyolysis, the primary defect may be intrinsic to the muscle fiber (metabolic myopathies) or extrinsic, such as excessive physical activity, environmental or therapeutic hyperthermia/hypothermia, endocrinopathies, and hydro-electrolytic alterations. 4 5 6 19 20 Rhabdomyolysis of intrinsic metabolic cause is related to genetic diseases compromising muscle energy supply, due to defects in the glycolytic (anaerobic metabolism), lipidic or oxidative pathways (aerobic metabolism). In metabolic myopathies, prolonged fasting may lead to rhabdomyolysis. Rhabdomyolysis also occurs in structural congenital myopathies, muscular dystrophies, and acquired myopathies, such as inflammatory myopathies. 10

Endocrine diseases associated with rhabdomyolysis are hypo/hyperthyroidism, diabetic ketoacidosis/nonketotic hyperosmolar coma, primary hyperaldosteronism, primary adrenal insufficiency, central diabetes insipidus, and pituitary diseases. Fluid/electrolyte imbalances that may lead to rhabdomyolysis are hypokalemia, hypocalcemia, hypophosphatemia, hypernatremia, hyperosmolarity, water intoxication and acidosis. Hypokalemia, such as that caused by chronic use of laxatives and potassium-depleting diuretics, induces rhabdomyolysis due to changes in vascular blood flow, causing relative ischemia. 4 Acute hypophosphatemia with secondary rhabdomyolysis can be precipitated by refeeding a malnourished patient, as phosphate is required for ATP synthesis. 4 Additionally, patients with acute intermittent porphyria can present rhabdomyolysis associated with hyponatremia. 21

Vascular changes can lead to rhabdomyolysis due to ischemia associated with autoimmune processes, arterial/venous obstruction, use of tourniquets, extracorporeal circulation, transplants, vascular prosthesis placement, and sickle-cell trait. 22 Electrical current injury can also lead to rhabdomyolysis in up to 10% of patients, resulting from excessive muscle activity and direct muscle injury. 5 6

Hypermetabolism, which causes an imbalance between energy supply/demand during strenuous physical activity, is a metabolic cause of rhabdomyolysis. It occurs in exertional heatstroke, characterized by changes in consciousness, hyperthermia, and rhabdomyolysis. In the United States, the annual incidence of exertional rhabdomyolysis among military personnel is 40:100,000 individuals, and it is more common among men, individuals under 19 years old, and African Americans. 23 Excessive muscle activity can cause rhabdomyolysis during infectious tetanus, status epilepticus, dystonia, or delirium tremens. In Mesoamerican nephropathy, a chronic kidney disease resulting from recurrent rhabdomyolysis in farmers, mainly those working in sugarcane fields, several factors contribute to rhabdomyolysis, such as physical activity, ambient heat, and dehydration. 24

### CLINICAL SYNDROMES COMMONLY ASSOCIATED WITH RHABDOMYOLYSIS

#### Neuroleptic malignant syndrome

Neuroleptic malignant syndrome is a rare idiosyncratic reaction found in 0.03 to 0.2% of patients exposed to neuroleptics, due to the dopamine receptor antagonism. 25 26 27 It has also been reported in patients using lithium and during the withdrawal of dopaminergic anti-Parkinsonian drugs. It occurs in all age groups, with a predominance of young adults and men (2:1). Mortality from NMS decreased from > 70% in the 1970s to less than 10% nowadays.

Patients often use neuroleptics for years, or even decades, before developing NMS. Neuroleptic malignant syndrome onset is gradual, between 24 and 72 hours, and is often linked to recent adjustments in dosage, combination/withdrawal of a related drug, or intravenous administration of neuroleptics. If detected and treated promptly, the condition lasts 7 to 10 days, with most patients being symptom-free after 30 days. Typical signs include altered level of consciousness (stupor, coma), cogwheel-type muscle rigidity, hyperthermia (often exceeding 41 °C), rhabdomyolysis, and autonomic dysfunction (sweating, tachycardia, tachypnea, blood pressure fluctuations, and arrhythmias) ( Table 2 ).

**Table 2:** Neuroleptic malignant syndrome: clinical and laboratory findings 25 26 27

Clinical/laboratory findings	Evidence
Positive antecedents	Neuroleptic introduction/change or levodopa interruption
Altered consciousness	Stupor or coma
Muscle rigidity	Cogwheel or lead-pipe rigidity
Temperature increase	Hyperthermia
Rhabdomyolysis	Creatine kinase or myoglobin elevation
Increased metabolism	Tachycardia, tachypnea
Autonomic dysfunction	Blood pressure oscillation, excessive sweating, loss of bladder control
Absence of other causes	Catatonia, central nervous system lesion, intoxication (e.g., cocaine), malignant hyperthermia, pheochromocytoma, sepsis, serotoninergic syndrome, thyrotoxicosis, withdrawal syndrome

A new NMS diagnostic criterion was recently developed and validated by a panel of experts. This criterion involves a list of signs and symptoms, each assigned a score, with a sensitivity of 69.6% and a specificity of 90.7% at a cutoff of 74 points ( Table 2 ). 26 27 Neuroleptic malignant syndrome treatment primarily includes the use of dopaminergic agents and the discontinuation of dopaminergic antagonist agents. Supportive measures involve active cooling and benzodiazepines for managing agitation. Non-depolarizing neuromuscular blockers may be employed to control muscle rigidity and related hyperthermia. During long-term treatment with dopaminergic agents, dantrolene can be administered in the first 24 hours (1 mg/kg, every 6 hours) for initial control of muscle rigidity. Electroconvulsive therapy has also been successfully used in cases that are refractory to other treatments.

#### Serotoninergic syndrome

Serotonin syndrome is caused by increased serotonin neurotransmitter (5-HT) levels. 28 29 Serotonin is synthesized from tryptophan. Enterochromaffin cells produce around 90% of serotonin, while less than 10% is stored in platelets. The remaining 1 to 2% of serotonin stores are found in the central nervous system.

Serotonin syndrome can be triggered by increased serotonin production (tryptophan supplementation) or increased release (amphetamines, cocaine, mirtazapine, and some opioids, such as meperidine and tramadol), reuptake inhibition (metoclopramide, selective serotonin reuptake inhibitors, and tricyclic antidepressants), and decreased metabolism (methylene blue and other monoamine oxidase and CYP inhibitors such as some antibiotics, antifungals and antivirals). It was also described after the use of valproate, ondansetron, and ginseng.

**Figure 2:**
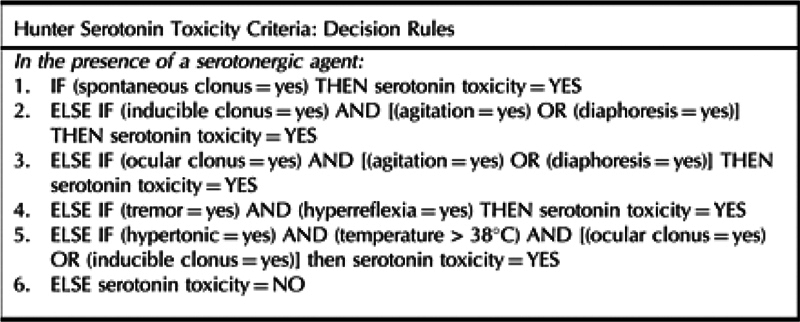
Hunter criteria for serotoninergic syndrome. 29 By permission of Oxford University Press.

The onset of the serotonin syndrome is typically sudden, occurring within 12 hours, depending on the dose. Symptoms such as agitation, hyperactivity, sweating, diarrhea, hyperreflexia, myoclonus, and ocular clonus may escalate to hyperthermia, seizures, mydriasis, coma, lower limb rigidity, and rhabdomyolysis. Diagnosis is supported by the Hunter criteria ( Figure 2 ). 29 Treatment includes discontinuation of the triggering agents and providing supportive care with benzodiazepines, active cooling, and hydration. In severe cases, 5-HT receptor antagonists like cyproheptadine, methysergide, and antipsychotics such as olanzapine and chlorpromazine can be beneficial.

#### Sympathomimetic syndrome

Sympathomimetic syndrome is induced by licit or illicit substances that act on adrenergic receptors. 30 31 The clinical presentation is variable, and the severity of the outcome depends on the drug's pharmacokinetic and pharmacodynamic profile. Intoxication by agents with sympathomimetic activity is associated with psychomotor agitation, aggressiveness, paranoia, hallucinations, confusion, anxiety, bruxism, delusions, convulsions, hyperthermia, rhabdomyolysis, tachycardia, hypertension, chest pain, myocarditis, Takotsubo cardiomyopathy, metabolic acidosis, and ventricular tachycardia/fibrillation.

Methamphetamines, amphetamines, cocaine and even prescription drugs such as methylphenidate and theophylline are associated with sympathomimetic syndrome. Recently, synthetic drugs have also been involved in severe forms of sympathomimetic syndrome. These synthetic drugs include phenylethylamines (MDMA, 2c-series of compounds, NBOMe, cathinones, ethylone) and provoke a hyperadrenergic state with variable effects on the serotonergic system. Tryptamines, in turn, are drugs that have similar molecular structures to those of N, N-dimethyltryptamine and lysergic acid diethylamide. They primarily affect the serotonergic system, with a lesser impact on the noradrenergic and dopaminergic systems.

Treatment consists of discontinuing the triggering agents and clinical support measures such as active cooling and hydration. In patients with severe hypertension, treatment with direct vasodilators (such as phentolamine, nitrates, and calcium channel blockers) is preferable to beta blockers due to the risk of coronary vasospasm.

#### Malignant hyperthermia

Malignant hyperthermia is triggered in genetically susceptible individuals by exposure to halogenated inhalational anesthetic agents and/or succinylcholine. 32 It is characterized by muscle rigidity, rhabdomyolysis, hyperthermia, hypermetabolism with hypercarbia, mixed acidosis and failure of multiple organs and systems. This autosomal dominant pharmacogenetic syndrome results from variants in the genes associated with the excitation-coupling system ( *RYR1, CACNA1S, STAC3* ), leading to increased calcium release.

Many patients susceptible to MH are completely asymptomatic until a crisis develops during anesthesia. But some of them present exercise intolerance, myalgia, cramps and muscle weakness. Malignant hyperthermia is allelic with some congenital myopathies, such as central core disease, multiminicore disease, and congenital fiber type disproportion. All these patients should be warned about the anesthetic risk and receive trigger-free anesthesia. Many countries have helplines and sites for orientation during malignant hyperthermia crises (emhg.org, mhaus.org, cedhima.unifesp.br).

For treatment, triggering agents should be discontinued. The patient must be hyperventilated with 100% oxygen. Dantrolene sodium should be used immediately after diagnosis, before the patient progresses to multiple organ failure. Dantrolene is a neuromuscular blocker that acts on the ryanodine type-1 receptor, preventing the release of calcium. Specific additional measures include cooling, bicarbonate, as well as treatment of arrhythmias and hyperpotassemia.

#### Anesthesia-induced rhabdomyolysis

Anesthesia-induced rhabdomyolysis was previously called a malignant hyperthermia-like syndrome. 33 34 This condition triggers sudden cardiac arrest in patients with subclinical myopathies, mainly in those exposed to succinylcholine and, less frequently, to halogenated agents. Most cases have been reported in patients with Duchenne muscular dystrophy.

It results from the upregulation of acetylcholine receptors (AChRs) and is characterized by the appearance of immature and neuronal forms of this receptor, in an extrajunctional location (outside the neuromuscular junction), and in greater numbers than normal. These atypical forms do not desensitize and are hypersensitive to membrane depolarization stimuli, causing excessive potassium outflow from the muscle fiber, which leads to muscle damage and hyperkalemia that can result in sudden cardiac arrest.

Upregulation of AChRs has also been observed in conditions of increased muscle catabolism, such as neuromuscular diseases in general, in lower or upper motor neuron injury, and during physical (e.g., intensive care unit patients) or chemical immobility (e.g., use of botulinum toxin). It is also seen in major burn patients, sepsis, and with prolonged use of neuromuscular blockers. There is no risk of hyperkalemia when neuromuscular blockade during anesthesia is performed with non-depolarizing drugs. However, using anticholinesterases to reverse the blockade should be avoided because of the risk of also activating atypical AChRs.

### MUSCULAR DISORDERS ASSOCIATED WITH RHABDOMYOLYSIS

#### Toxic myopathies

A toxic myopathy is characterized by acute or subacute manifestation of muscular symptoms/signs, such as muscle weakness, myalgia, creatine kinase (CK) elevation, or myoglobinuria. 35 36 37 38 Although most clinicians are aware of the myotoxic potential of many drugs, the exact mechanisms and the patients at risk have not been well understood previously. The pathogenic bases can be multifactorial. Toxic myopathies are often a diagnosis of exclusion, as the differential diagnosis for muscle symptoms can be quite broad.

Many substances, including commonly prescribed medications, can produce adverse effects on muscle ( Table 1 ). They are potentially reversible at early stages, so clinicians should recognize toxic myopathies early in their course to initiate therapy and prevent irreversible damage.

Cholesterol-lowering drugs are the most prescribed drugs that have been reported to cause myopathy in recent years. Besides toxic necrotizing myopathy caused by statins, which stops with discontinuation of the medication, statins also trigger an autoimmune myopathy that progresses for months after statin discontinuation, named immune-mediated necrotizing myopathy. 36 It is induced by the antibody anti-HMGCR.

Immune checkpoint inhibitors are antibodies that bind the programmed cell death 1 (PD-1) receptors found on the surface of T-cells. They can cause severe acute weakness, within 1 day of starting these therapies, or a delayed myopathy by up to 19 weeks. Associated bulbar or ocular dysfunction can mimic the symptoms of myasthenia gravis.

Sometimes, the occurrence of rhabdomyolysis after the use of a small amount of a myotoxic substance may reveal subclinical metabolic myopathy (e.g., fatty acid beta oxidation defects and mitochondrial myopathies). The use of the RHABDO criteria helps to select patients who deserve further investigation for underlying genetic diseases: R - recurrent episodes, H - hyperCKemia for a prolonged period, A - accustomed physical activity that cannot sufficiently explain the severity of the event, B - blood CK > 50x upper limit of normal, D - drug/medication that are insufficient to explain the severity, O - other affected family members/other symptoms. 39 In patients who met at least one of these criteria, almost 40% had a genetic disease underlying the rhabdomyolysis. 39

#### Idiopathic inflammatory myopathies

Idiopathic inflammatory myopathies represent a specific subset of disorders within the broader category of inflammatory myopathies. 40 41 42 They comprise distinct entities that for many years have been classified as polymyositis, dermatomyositis, and inclusion body myositis (IBM). Recently, the classification has been modified based on subgroups with specific clinical symptoms, serum auto-antibodies, and morphological aspects, being described now four main subtypes: dermatomyositis, sporadic IBM, immune-mediated necrotizing myopathies, and the anti-synthetases group. All of them can present as rhabdomyolysis. 43 44 45

Characteristic morphological phenotypes in muscle biopsies allow an accurate diagnosis of myositis sub-entities. The autoantibodies are represented by two types: myositis-specific antibodies (MSAs) and myositis-associated antibodies (MAAs), and there is a strong correlation between serum autoantibodies and muscle pathology. The association among clinical features, MSAs/MAAs, and myopathology can contribute to early diagnosis, prognosis, and a better therapeutic result.

#### Muscular dystrophies

Muscular dystrophies represent a heterogeneous group of genetic diseases characterized by degeneration of muscle fibers and progressive muscle weakness. These disorders are caused by a deficiency of proteins essential for maintaining muscle fiber integrity during the contraction process, as occurs in dystrophin protein deficiency, leaving the muscle fiber vulnerable to injury. Therefore, additional muscle damage can trigger myoglobinuria in these patients. 46 47 48

Many of these patients present pseudometabolic symptoms such as fatigue, exercise intolerance, and cramps, sometimes preceding the onset of muscle weakness, which leads to the erroneous diagnosis of metabolic myopathy. 48 Exercise intolerance and myoglobinuria are manifestations described in patients with different forms of muscular dystrophy. The ones most associated with myoglobinuria are linked to dystrophin (Duchenne or Becker muscular dystrophies), fukutin-related protein (FKRP), and anoctamin-5. 39 48 49 50

In a study by Lahoria and Milone (2016), 13 unrelated patients were identified in a cohort with genetically confirmed muscular dystrophy in whom rhabdomyolysis was the presenting or main clinical manifestation. 48 Fukutin-related protein -related muscular dystrophy (n = 6) was the most common diagnosis, followed by anoctaminopathy-5 (ANO5) (n = 3), calpainopathy-3 (n = 2), and dystrophinopathy (n = 2). Exercise and fever were common triggers; however, rhabdomyolysis was unprovoked in three patients. 48 Rhabdomyolysis was reported in an additional case of ANO5. 51

Episodes compatible with exercise-induced myoglobinuria appear to be frequent in FKRP-related muscular dystrophy. In the study of Lindberg et al. (2012), 5 patients with FKRP-related muscular dystrophy from a cohort of 14 patients reported recurrent episodes of dark urine and myalgia after exercise, and in 3 of them, this was the only symptom for several years. 49 In another cohort of FKRP-related muscular dystrophy, from 26 patients, 7 (27%) had at least 1 episode of myoglobinuria. 52 In three patients, the diagnosis of muscular dystrophy was made after the episodes of myoglobinuria triggered by exercise. General anesthesia also triggered one episode of myoglobinuria. 52

Among the main triggering factors are exercise, fever, and anesthesia with succinylcholine and/or inhalation of halogenated anesthetic agents, mostly in Duchenne and Becker muscular dystrophies. 53 Bisphosphonates are another potential cause of myoglobinuria in patients with Duchenne muscular dystrophy. 54 55

Mutations in the caveolin gene (CAV3) have also been associated with rhabdomyolysis. 46 47 In a series of eight patients from seven families, seven had myalgia, seven had exercise intolerance, and two developed rhabdomyolysis. 46

Other forms of muscular dystrophies that may eventually manifest with myoglobinuria include sarcoglycanopathies, dysferlinopathy, Fukuyama-type congenital muscular dystrophy and myotonic dystrophy. 56 57 58 59 60 61 In many of these cases, episodes of myoglobinuria occurred even before the onset of evident muscle weakness. 58 59 These reports suggest that a diagnosis of muscular dystrophy should be considered in cases of rhabdomyolysis, especially those induced by exercise, even if muscle weakness is not evident.

#### Ion channel diseases (channelopathies)

Ion channels are responsible for modulating the electrical activity of the sarcolemma. Therefore, channelopathies are considered disorders of muscle fiber excitability, which can cause paralysis (due to membrane inexcitability) or myotonia (due to membrane hyperexcitability) ( Table 3 ).

**Table 3:** Summary of affected genes and electrophysiological consequences in muscle channelopathies

Affected gene	Electrophysiological consequence	Associated disease
*SCN4A*	Loss of function: failure in the rapid inactivation of the channel. Sustained entry of sodium, leading to persistent sarcolemma depolarization, which inactivates normal sodium channels, causing paralysis Gain of function: increase in excitability that manifests as myotonia and hypertrophy, due to constant activity	Hyperkalemic PP Paramyotonia congenital Sodium channel myotonia Hypokalemic PP
*KCNJ2*	Reduced potassium entry into the cell, causing difficulty in membrane repolarization in muscle, heart and bone. In addition, Kir2.1 is believed to be critical for osteoblastogenesis, which would explain the presence of dysmorphic alterations in this syndrome	Andersen-Tawil syndrome
*KCNJ18*	Reduced Kir2.6 currents, with some of these effects being dependent on the thyroid hormone T3. Under normal conditions, Kir2.6 channels are activated by T3 in normal muscle to maintain the resting potential, which would protect against weakness induced by excess T3. In the case of the KCNJ18 mutation, the mutant has a dominant-negative effect on normal Kir2.6 and Kir2.1, which would cause weakness	Thyrotoxic PP
*CACNA1S*	Aberrant pore current, causing paradoxical depolarization in conditions of low serum potassium and consequent membrane inexcitability	Hypokalemic PP
*ClC-1*	Resting membrane potential of skeletal muscle is not sustained which predisposes to autonomous depolarizations. In Thomsen's disease, the mutated subunit of the channel exerts a dominant-negative effect over the normal subunit, which does not occur in Becker's disease. For the manifestation to occur, the mutation must cause a reduction in channel conductance of more than 50%.	Autosomal dominant myotonia congenita (Thomsen's disease) Autosomal recessive myotonia congenita (Becker's disease)

Abbreviations: CACNA1S, calcium voltage-gated channel subunit alpha1S; ClC-1: chloride voltage-gated channel 1; KCNJ2, Potassium Inwardly Rectifying Channel Subfamily J Member 2; KCNJ18, inward rectifier potassium channel 18 gene; Kir2.1, inwardly rectifying potassium channel; K _ir_ 2.6, inward rectifier potassium channel 18 - encoded by the KCNJ18 gene; PP, periodic paralysis; SCN4A, sodium voltage-gated channel alpha subunit 4.

These diseases can be grouped into those that present periodic paralysis (PP), myotonia or both. Periodic paralysis is characterized by recurrent episodes of flaccid paralysis that can be generalized or localized, lasting from hours to a few days, with complete recovery of muscle strength. Myotonia is characterized by difficulty in relaxing the muscles after a voluntary contraction. The channelopathies that present with PP are the hypokalemic PP, thyrotoxic PP, and Andersen-Tawil syndrome. Sodium channel myotonia and myotonia congenita are in the group that present with myotonia, and hyperkalemic PP and paramyotonia congenita can present both manifestations, with periodic paralysis being predominant in the former. Both myotonias and periodic paralysis have been associated with rhabdomyolysis. 19 62

Mutations in *SCN4A* (sodium channel) have been identified in patients with skeletal muscle channelopathies, including hyperkalemic PP, paramyotonia congenita, sodium channel myotonia and more rarely hypokalemic PP. 63 Alterations in potassium channels have been found in Andersen-Tawil syndrome, a disease characterized by the triad PP (hypokalemic or hyperkalemic), cardiac arrhythmia and dysmorphism; and thyrotoxic PP. Hypokalemic PPs are caused, in most cases, by mutations in the *CACNA1S* (calcium channel), also involved in some cases of malignant hyperthermia susceptibility. Alterations in the *ClC* -1 (chloride channel) have been associated with congenital myotonias: Thomsen's disease (autosomal dominant inheritance) and Becker's disease (autosomal recessive inheritance). Electrophysiological consequences ( Table 3 ) and clinical manifestations depend on the channel affected and the location of the mutation, with loss or gain of function resulting in weakness or myotonia. 64 65 66 67

#### Glycogen storage diseases (GSDs)

Glycogen storage diseases group encompasses several rare conditions related to inborn errors of metabolism resulting from enzyme deficiencies that affect glycogen synthesis or breakdown. 68 69 70 71 72 73 74 There is involvement of multiple organs, mostly muscles and liver, where glycogen is abundant, but also the heart, kidney, and brain.

The synthesis and degradation of glycogen involve separate sets of enzymes, functioning irreversibly: the degradation process is not the reverse of synthesis. 68 The absence of just one enzyme can compromise the synthesis/degradation, or cause glycogen molecule abnormalities. Additionally, some enzymes exhibit different isoforms in various organs, resulting in variable clinical manifestations. The severity of the disease is inversely related to the enzyme residual activity

Glycogen storage diseases can present at any age, with an incidence of approximately 1:20,000 to 43,000 live births. 72 Certain GSD enzyme defects cause muscle impairment, which may manifest as fatigue, myalgia, cramps, exercise intolerance, muscle weakness, and/or muscle contractures. Glycogen storage diseases are a classic cause of rhabdomyolysis triggered by intense, brief physical activity. Electromyography and muscle biopsy can show a myopathic pattern but should be postponed until complete recovery from an event of rhabdomyolysis. 19

There are 12 GSD with muscle involvement ( Table 4 ). Rhabdomyolysis is most associated with the deficiencies of phosphorylase (McArdle's disease, the most common 73 ), phosphofructokinase, phosphoglycerate mutase, lactate dehydrogenase, and aldolase. 72 73 74 Diagnosis can be made primarily by a genetic molecular test. 72 73 74 Muscle biopsy may be normal or show abnormal glycogen accumulation, especially in type-2 fibers; additionally, biochemistry and histochemistry can show decreased activity or absence of the specific enzyme activity. 68

**Table 4:** Main glycogen storage diseases with muscle involvement 68 69 72 73 74

Disease number	Enzyme/Transporter	Clinical picture	Laboratory hallmarks
II	Acid alpha-glucosidase (Pompe's disease)	Skeletal and cardiac myopathy	
IIIa	Glycogen debranching enzyme (Cori/Forbes disease)	Skeletal and cardiac myopathy, hepatopathy	Dyslipidemia Hypoglycemia
IV	Glycogen branching enzyme : α-1,4-glucan-6-glucosyl transferase (Andersen's disease or amylopectinosis)	Skeletal and cardiac myopathy, hepatopathy, neuropathy, central/peripheral nervous system involvement	Hypoglycemia
V	Phosphorylase (McArdle's disease)	Exercise intolerance, cramps, myalgia, rhabdomyolysis, “second wind”*	Absence of increase of serum lactate after forearm exercise test
VII	Phosphofructokinase deficiency (Tarui's disease)	Exercise intolerance, cramps, myalgia, rhabdomyolysis, cardiopathy, seizures, gout, “out of wind”#	Hemolytic anemia, absence of increase of serum lactate after forearm exercise test
IX-b	Phosphorylase beta-kinase	Mild/absent muscle complaints, hepatopathy	Dyslipidemia, hypoglycemia
IX-d	Phosphorylase alfa-kinase	Skeletal myopathy	
X	Phosphoglycerate mutase	Exercise intolerance, cramps, myalgia, rhabdomyolysis, gout	
XI	Lactate dehydrogenase	Exercise intolerance, myalgia, skin lesions, rhabdomyolysis	↑ pyruvate and serum lactate after forearm exercise test
XII	Aldolase	Skeletal myopathy, rhabdomyolysis, brain disease	Hemolytic anemia
XIII	Enolase	Skeletal myopathy, exercise intolerance, myalgia	Normal serum lactate after forearm exercise test
XV	Glycogenin	Skeletal and cardiac myopathy	

Notes: *improvement in performance after some minutes of exercise. # worsening of performance when sugar is taken before exercise.

#### Fatty acid beta-oxidation defects and mitochondrial myopathies

Mitochondria are responsible for many crucial cell functions, such as the beta oxidation of fatty acids and the oxidative phosphorylation for energy production. Rhabdomyolysis is mostly described in association with deficiencies of carnitine palmitoyl-transferase II, acyl-coenzyme-A-transferase, succinate dehydrogenase, cytochrome-c oxidase, and coenzyme Q10.

Fatty acid beta oxidation defects result from a deficiency of enzymes involved in the degradation of fatty acids for energy production and are characterized by recurrent rhabdomyolysis, hypoketotic hypoglycemia, arrhythmia, cardiopathy (hypertrophic or dilated), and myopathy that can be expressed as exercise intolerance, fatigue, myalgia, and muscle weakness. 75 Rhabdomyolysis can be precipitated not only by fasting but also by cold, stress, febrile illness, and prolonged exercise.

Mitochondrial diseases are multisystemic diseases characterized by impairment of oxidative phosphorylation, affecting mostly tissues with higher metabolic activity, such as the nervous system (mitochondrial encephalopathy), and skeletal/cardiac muscles (mitochondrial cardiopathy/myopathy). The main complaints in mitochondrial myopathies are exercise intolerance, myalgia and muscle weakness, frequently involving the extrinsic ocular musculature. Exercise-induced rhabdomyolysis has been described in isolated patients with variants in the mtDNA *MT-CO2* , 76 *MT-ND1* with complex 1 deficiency, 77 ferredoxin-2, 78 and thymidine kinase-2 deficiency. 79

The investigation includes serum lactate/pyruvate ratio, acylcarnitine analysis (plasma/lymphocyte/cultured fibroblasts), plasma/urinary profile of amino/organic acids, muscular biochemistry/histochemistry, and molecular study.

### TREATMENT

After the acute necrosis phase, most patients with rhabdomyolysis experience muscle recovery. Clinical management is based on three strategies: controlling the underlying cause, fluid resuscitation, and managing electrolyte abnormalities, along with other complications. 4 80 Removing the offending factor prevents further damage, but it may not avoid complications once a large amount of muscle components has already been released into the bloodstream. Additionally, secondary ischemia associated with compartment syndrome can cause rhabdomyolysis to persist even without the original stimulus.

#### Acute kidney injury and fluid/hydroelectrolytic imbalances

The initial strategy is volume replacement, which can lower the risk of AKI. 4 Forced diuresis with diuretics (e.g. mannitol) and urinary alkalinization using bicarbonate have failed to show benefit in preventing AKI, and their effectiveness has been questioned. 4 The presence of anuric AKI may hinder aggressive fluid resuscitation and can lead to early renal replacement therapy.

It is also necessary to correct the secondary hydroelectrolytic imbalances, particularly hyperkalemia, hyperphosphatemia and acidosis. Hyperkalemia is managed with insulin solution combined with glucose, beta-adrenergics, and diuretics. Phosphorus chelators are used in hyperphosphatemia. Calcium reposition is controversial, as initial hypocalcemia is followed by hypercalcemia in the progression of rhabdomyolysis. 4 Renal replacement therapy can be used to treat severe hyperkalemia, hyperphosphatemia, water intoxication, uremia, and acidosis.

#### Hyperthermia

Hyperthermia caused by abnormal muscle activity does not respond to antipyretic medications and requires external cooling with ice packs, as well as perfusion of cavities and intravenous administration of cooled solutions. Non-depolarizing neuromuscular blocking agents should be used in cases of muscle rigidity induced by central nervous system dysfunction.

#### Compartment syndrome

Surgical intervention is necessary in cases of compartment syndrome, typically involving fasciotomy to relieve intracompartmental pressure. However, due to the risk of secondary infection, surgery is advised only when the mean intramuscular arterial pressure exceeds 30 mmHg.

### PREVENTION

Monitoring serum CK and serum/urinary myoglobin levels in risk situations enables early detection/treatment. In patients at risk of rhabdomyolysis, a preoperative CK measurement acts as a useful parameter and helps differentiate chronic increases from acute elevations related to anesthesia/surgery.

In the preoperative assessment, neurologists and anesthesiologists must identify risk factors for rhabdomyolysis and manage them individually. During surgery positioning, care should be taken to avoid compressing muscle groups. In vascular surgeries, clamping should be as short as possible or intermittent to prevent rhabdomyolysis related to the ischemia-reperfusion process. Patients with metabolic myopathy should be advised to avoid excessive muscle activity and prolonged fasting before surgery. For those with beta oxidation defects and mitochondrial myopathies, it is recommended to avoid propofol, reduce psychological and physical stress, and admit the patient early for intravenous hydration and glucose intake during the preoperative fast.

Patients diagnosed with myopathy, regardless of the cause, should not receive succinylcholine. Halogenated drugs should be avoided in ryanodinopathies, particularly MH and myopathies with cores. In patients with Duchenne/Becker muscular dystrophy, in whom halogenated agents can trigger AIR, the risks and benefits of using the halogenated agent should be carefully considered. When necessary, it should be administered at the lowest effective dose and for the shortest duration possible.

Patients previously admitted to intensive care units are at risk of rhabdomyolysis due to the combination of factors such as fluid/electrolyte imbalances, polytherapy, prolonged immobilization, and infections. Other groups at risk include patients on statins who may have chronic subclinical myopathies, athletes admitted with exertional heat illness, and users of illegal drugs admitted to the emergency department. Particular attention should be given to cases of acute alcohol poisoning, which can lead to acute rhabdomyolysis and myocarditis.

In conclusion, the sporadic occurrence of rhabdomyolysis, its considerable clinical variability, and differential diagnosis require healthcare professionals to maintain a high level of diagnostic vigilance. The present narrative review highlighted the main syndromes and muscular disorders associated with rhabdomyolysis, with respective relevant clinical tools. Future research could focus on measures to increase identification of rhabdomyolysis and its subjacent etiology, as well as to decrease the complication/mortality rate.
